# Antarctic Moss Multiprotein Bridging Factor 1c Overexpression in Arabidopsis Resulted in Enhanced Tolerance to Salt Stress

**DOI:** 10.3389/fpls.2017.01206

**Published:** 2017-07-11

**Authors:** Hemasundar Alavilli, Hyoungseok Lee, Mira Park, Byeong-ha Lee

**Affiliations:** ^1^Department of Life Science, Sogang University Seoul, South Korea; ^2^Division of Life Sciences, Korea Polar Research Institute Incheon, South Korea

**Keywords:** stress tolerance, salt stress, MBF1c, RNA sequencing, Antarctic moss, *Polytrichastrum alpinum*

## Abstract

*Polytrichastrum alpinum* is one of the moss species that survives extreme conditions in the Antarctic. In order to explore the functional benefits of moss genetic resources, *P. alpinum* multiprotein-bridging factor 1c gene (*PaMBF1c*) was isolated and characterized. The deduced amino acid sequence of PaMBF1c comprises of a multiprotein-bridging factor (MBF1) domain and a helix-turn-helix (HTH) domain. *PaMBF1c* expression was induced by different abiotic stresses in *P. alpinum*, implying its roles in stress responses. We overexpressed *PaMBF1c* in Arabidopsis and analyzed the resulting phenotypes in comparison with wild type and/or Arabidopsis *MBF1c* (*AtMBF1c*) overexpressors. Overexpression of *PaMBF1c* in Arabidopsis resulted in enhanced tolerance to salt and osmotic stress, as well as to cold and heat stress. More specifically, enhanced salt tolerance was observed in *PaMBF1c* overexpressors in comparison to wild type but not clearly observable in *AtMBF1c* overexpressing lines. Thus, these results implicate the evolution of *PaMBF1c* under salt-enriched Antarctic soil. RNA-Seq profiling of NaCl-treated plants revealed that 10 salt-stress inducible genes were already up-regulated in *PaMBF1c* overexpressing plants even before NaCl treatment. Gene ontology enrichment analysis with salt up-regulated genes in each line uncovered that the terms lipid metabolic process, ion transport, and cellular amino acid biosynthetic process were significantly enriched in *PaMBF1c* overexpressors. Additionally, gene enrichment analysis with salt down-regulated genes in each line revealed that the enriched categories in wild type were not significantly overrepresented in *PaMBF1c* overexpressing lines. The up-regulation of several genes only in *PaMBF1c* overexpressing lines suggest that enhanced salt tolerance in *PaMBF1c*-OE might involve reactive oxygen species detoxification, maintenance of ATP homeostasis, and facilitation of Ca^2+^ signaling. Interestingly, many salt down-regulated ribosome- and translation-related genes were not down-regulated in *PaMBF1c* overexpressing lines under salt stress. These differentially regulated genes by *PaMBF1c* overexpression could contribute to the enhanced tolerance in *PaMBF1c* overexpressing lines under salt stress.

## Introduction

In the Antarctic landscape, mosses constitute the dominant flora. They are capable of coping with multiple abiotic stress factors such as low temperatures, high radiation, high salts, strong winds and prolonged desiccation, and unpredictable cycles of freezing and thawing (Turetsky et al., 2012; Zuniga-Gonzalez et al., 2016). *Polytrichastrum alpinum* (Hedw.) G.L.Sm., also known as alpine haircap moss, is distributed over a large area of arctic, sub-arctic and montane temperate regions (Bhattarai et al., 2009; Bell and Hyvonen, 2010; Victoria et al., 2013). Despite its strong stress tolerance, few reports till date have described the utilization of moss genetic resources in crop improvement.

Multiprotein bridging factor 1 (MBF1) was first purified from posterior silk gland extracts of *Bombyx mori* (Li et al., 1994). MBF1 proteins are highly conserved from archaea to humans. Each of the three Arabidopsis *MBF1* paralogs could restore *MBF1* functions in *mbf1* deficient yeast (*mbf1*Δ) (Tsuda et al., 2004). In addition, the *mbf1*Δ yeast were also rescued by expressing human or silkworm *MBF1* (Takemaru et al., 1997), suggesting conservation of *MBF1* gene function. MBF1 proteins function as a non-DNA binding transcriptional co-activators (Aravind and Koonin, 1999; Kabe et al., 1999) that are involved in diverse physiological and developmental processes (Brendel et al., 2002; Liu et al., 2003).

In plants, *MBF1* genes are known to be involved in abiotic and biotic stress tolerance. *MBF1* expression in *Solanum tuberosum* (*StMBF1*) is induced by wounding (Godoy et al., 2001) and pathogen attack in tubers (Arce et al., 2006). Expression levels of *Vitis vinifera MBF1* (*VvMBF1*) were increased in leaf tissues in response to ABA and dehydration stress, and overexpression of *VvMBF1* in Arabidopsis resulted in enhanced drought stress tolerance (Yan et al., 2014); also, ectopic expression of *Triticum aestivum MBF1c* (*TaMBF1c*) in rice improved its thermal tolerance under vegetative and reproductive stages (Qin et al., 2015). However, *MBF1* overexpression does not always appear to result in improved stress tolerance in plants. When the *AtMBF1a* and *AtMBF1b* homolog, *Capsicum annum MBF1* (*CaMBF1*) was overexpressed in Arabidopsis, the resulting transgenic plants produced large leaves but displayed reduced tolerance to abiotic stress (Guo et al., 2014).

The model plant *Arabidopsis thaliana* has three *MBF1* paralogs – *AtMBF1a, AtMBF1b*, and *AtMBF1c*. Phylogenetic analysis suggests that *AtMBF1a* and *AtMBF1b* are more closely related, while *AtMBF1c* belongs to a separate group (Tsuda and Yamazaki, 2004). Overexpression of the *AtMBF1a* gene enhanced salt tolerance, glucose insensitivity and fungal resistance in transgenic Arabidopsis plants (Kim et al., 2007). Compared to the other two Arabidopsis *MBF1*s, *AtMBF1c* was highly induced in response to pathogen infection, dehydration, high salt, methyl viologen, hydrogen peroxide (H_2_O_2_), and heat treatment (Rizhsky et al., 2002; Tsuda and Yamazaki, 2004; Suzuki et al., 2005). The Arabidopsis *mbf1* triple knock-down mutant (*mbf1 abc-*) was hypersensitive to oxidative and osmotic stress agents such as methyl viologen, H_2_O_2_ and high concentrations of sorbitol. These *mbf1 abc-* mutant stress-sensitive phenotypes were either partially or fully restored by *AtMBF1c* cDNA overexpression, implicating the predominance of *AtMBF1c* gene function in stress tolerance (Arce et al., 2010). Overexpression of the *AtMBF1c* gene in Arabidopsis also enhanced tolerance to bacterial infection as well as to heat and osmotic stress (Suzuki et al., 2005). In fact, functionality of the *AtMBF1c* gene is well established in plant heat stress response; it controls heat stress-related gene expression to improve basal heat tolerance during heat stress (Suzuki et al., 2011).

In order to investigate the functional benefits of moss genetic resources, we have isolated the stress responsive *PaMBF1c* gene from a *P. alpinum* cDNA library and examined its functions under salt and other abiotic stress by overexpression and RNA-Seq profiling.

## Materials and Methods

### Conserved Domain Analysis and Phylogenetic Analysis

The MBF protein homologs sequences from diverse species were retrieved from GenBank database and Phytozome database^1^ (Goodstein et al., 2012) with BlastP using PaMBF1c amino acid sequences as a query. Multiple sequence alignment was performed using ClustalW (Thompson et al., 1994; Kumar et al., 2016). Conserved domains in each sequence were identified using NCBI conserved domain finder^2^. The phylogenetic tree was constructed with MEGA7 software (Thompson et al., 1994; Kumar et al., 2016) from the data sets by using the maximum likelihood method based on the JTT matrix-based model. The initial tree for the heuristic search was obtained by applying the neighbor-joining method to a matrix of pair-wise distances, estimated using a JTT model. Supports for internal branches were tested by bootstrap analyses of 1000 replications.

### Plant Material and Growth Conditions

*Polytrichastrum alpinum* (Hedw.) G.L.Sm. samples were collected from the King Sejong Antarctic station (62°14′29′′S; 58°44′18′′W), at the Barton Peninsula of King George Island. *In vitro* culture was carried out on BCD solid media (Ashton and Cove, 1977), which are successfully being used in a model moss *Physcomitrella patens* culture (Du et al., 2016), in a growth room operating at 23°C with 16-h light/8-h dark light cycle with a light intensity of 150 μmol m^-2^S^-1^.

The plates were placed in a growth chamber that operates at 22 ± 1°C and 70% relative humidity with continuous light (80–100 μmol m^-2^S^-1^). The seeds planted on soil (Sungro propagation mixture, Canada) were maintained in a controlled growth chamber that operates with 16-h light/8-h dark cycle. All seeds were stratified for at least 2 days at 4°C before being transferred to the growth chamber. When necessary, seedlings raised on MS were transferred to soil pots in a growth chamber at 22 ± 1°C and 50–70% relative humidity programmed with 16-h light/8-h dark light cycle (the light intensity of 80–100 μmol m^-2^S^-1^).

### Cloning of *PaMBF1c* Gene and Generation of Transgenic Arabidopsis Plants

*Polytrichastrum alpinum* first strand cDNA was synthesized from total RNA with MMLV reverse transcriptase (Enzynomics, South Korea) and oligo(dT) primer. Full-length *PaMBF1c* coding sequences were amplified using gene specific primers (Supplementary Table S1). The resulting PCR products were cloned into the pENTR/D/TOPO entry vector (Invitrogen, United States) and sequenced using M-13 primers. After sequencing confirmation, the entry plasmids were LR-recombined with the gateway compatible binary destination vector, pMDC32 (Curtis and Grossniklaus, 2003), which resulted in the overexpression construct, pMDC32-35s:*PaMBF1c.* The construct was then transferred into *Agrobacterium tumefaciens* strain GV3101 via electroporation. Agrobacterium-mediated transformation of *Arabidopsis thaliana* (Col-0) with the *PaMBF1c* gene was performed through the floral dipping method (Clough and Bent, 1998). For selection of *PaMBF1c*-OE lines, T1 seeds were harvested from floral-dipped plants and selected on MS/agar plates containing hygromycin (25 μg/mL). Using hygromycin selection and transgene detection, homozygotes for the *PaMBF1c* transgene of two independent events were selected at T2 generation after segregation analysis at T3 generation (*PaMBF1c-*OE1 and *PaMBF1c-*OE2). Hygromycin resistance from each line at T2 generation was segregated to a 3:1 ratio of resistant-to-sensitive, indicating a single locus of insertion.

### Stress Treatment and Analysis of Stress Tolerance in Plants

*Polytrichastrum alpinum* gametophores were transferred onto fresh agar plates of BCD medium containing mannitol (150, 300 mM) or NaCl (75, 150 mM), respectively, and incubated at 15°C for 6 h. Heat treatment was carried out by transferring colonies grown at 15°C to chambers of 37 or 42°C for 2 h.

For *Arabidopsis thaliana* germination experiments, at least 100–120 seeds of each genotype were planted in a media with or without stress agents and germination trends were recorded from the day after planting until all the seeds in control plates were germinated. The number of germinated seeds was expressed as the percentage of total number of seeds plated. For root growth experiments, the seeds were planted in vertical MS plates with 0.6% gelrite and allowed to grow for 4 days. Seedlings with a 1–1.5 cm long root were transferred onto a second MS vertical plate supplemented with different concentrations of salts or stress agents. For survival index under NaCl and LiCl, the shoot phenotypes of seedlings were observed every day after transferring seedlings from the control medium. Hypocotyl elongation under heat stress was assessed as previously described (Hong and Vierling, 2000). The malondialdehyde (MDA), chlorophyll, and anthocyanin content in control and stress treated plants were also determined as previously described (Heath and Packer, 1968; Lichtenthaler, 1987; Neff and Chory, 1998).

### Measurement of Electrolyte Leakage

Fully expanded fourth or fifth rosette leaf with petiole from 3-week-old seedlings was placed in a 15 mL test tube containing 100 μL of deionized water and placed at 0°C in a refrigerated circulating bath (Gaon Science Instrument, South Korea). The remaining steps were carried out according to methods described previously (Lee et al., 2002). Conductivity was measured using a conductivity meter (Control Company, United States) before and after autoclaving, and electrolyte leakage was expressed as a percentage of conductivity before autoclaving over conductivity after autoclaving.

### Gene Expression Analysis

Total RNAs were isolated from plant materials using an RNA purification kit (NanoHelix, South Korea) and treated with RNase free DNase I (Qiagen, Germany). For semi-quantitative one step RT-PCR, total RNAs were added to the Hi-pure one step RT-PCR master mix (Genepole, South Korea) and the PCR reaction was performed according to manufacturer instructions. *Protein phosphatase 2A* (At1g13320) was used as an internal control. Quantitative real-time PCR was performed using KAPA SYBR FAST qPCR kit (Kapa Biosystems, United States) according to manufacturer instructions and run on the ABI 7500 system (Applied Biosystems, United States). The relative expression of *PaMBF1c* in *P. alpinum* was calculated by normalizing expression values with those of the housekeeping gene *PaTubulin*. For the Arabidopsis qRT experiment, *AtClathrin* gene (At4g24550) was used as an internal control. The ΔΔCt method was adapted to calculate relative gene expression (Livak and Schmittgen, 2001). The primer pairs used for amplification are shown in Supplementary Table S1.

### RNA Sequencing

RNA-sequencing was carried out using total RNA from 2-week-old WT, two independent *PaMBF1c*-OE lines (two events) and two independent *AtMBF1c*-OE lines (two events) under normal and salt stressed conditions (300 mM NaCl, 6 h). We used this salt stress condition because 300 mM NaCl treatment for 4–6 h in Arabidopsis brought about maximum expression of a very well characterized stress inducible gene, *RD29A* (Ishitani et al., 1997). Total RNAs were extracted from at least 25–30 plants of each genotype and treatment using Pure Helix total RNA purification kit (NanoHelix, South Korea) and RNase free DNase I (Qiagen, Germany). Three different biological replicates were prepared. The integrity and concentration of RNA was determined using Bioanalyzer (RIN > 6) and Qubit^®^ RNA BR assay kit (Life Technologies, United States). To construct the sequencing library, 1.5 μg of total RNA of each sample was used as input for the TruSeq RNA sample prep kit v2 (Illumina, United States). The libraries were validated and quantified by Bioanalyzer and qPCR quantification method, and then multiplexed with equal ratio and loaded on the flowcell of the Illumina MiSeq Reagent Kit v3 (150 cycles). Afterward, sequencing was performed on a MiSeq Sequencer system (Illumina, United States) and total 3 Gb (40M paired end reads) of sequencing data was generated (Q_30_ > 98%). The RNA-Seq data have been deposited to the Sequence Read Archive (SRA^3^) under accession number SRP110226.

### Transcriptomic Data Analysis

Basically all analyses were performed using the CLC Genomics Workbench v7 module (Qiagen, Germany). After quality and adapter trimming, raw reads were mapped to the Arabidopsis gene model annotation file of the Gene Ontology Consortium (released at August 8th, 2014). The expression values were measured in FPKM (Fragments per Kilobase of exon model per Million mapped reads) normalized values in gene level (Mortazavi et al., 2008). For statistical analysis, *t*-test and Baggerley’s test were performed using original and normalized read counts, and several relevant values for analysis (*p*-value, FDR corrected *p*-value, test-statistic, etc.) were calculated using the “multi-group comparison” option of the program. Through statistical analysis, differentially expressed genes were determined from a cutoff value (*p*-value < 0.05, corrected *p*-value of FDR < 0.05 and absolute value of fold change > 1.5) from pairwise comparison of normalized FPKM values between samples. Gene ontology (GO) enrichment analysis was performed using the PANTHER overrepresentation test (PANTHER version 10^4^) (Mi et al., 2013, 2016). *Arabidopsis thaliana* was selected as a reference organism with default settings and Bonferroni correction for multiple testing was used (*p* < 0.05).

### Statistical Analysis

All statistical comparisons between variants were determined by ANOVA (analysis of variance) and least significant differences (LSD) between variants were calculated using Statistix 8.1 computation software. Statistically significant mean values were denoted as ^∗^ (*p*-value ≤ 0.05).

## Results

### Sequence and Phylogenetic Analysis of PaMBF1c

**FIGURE 1:**
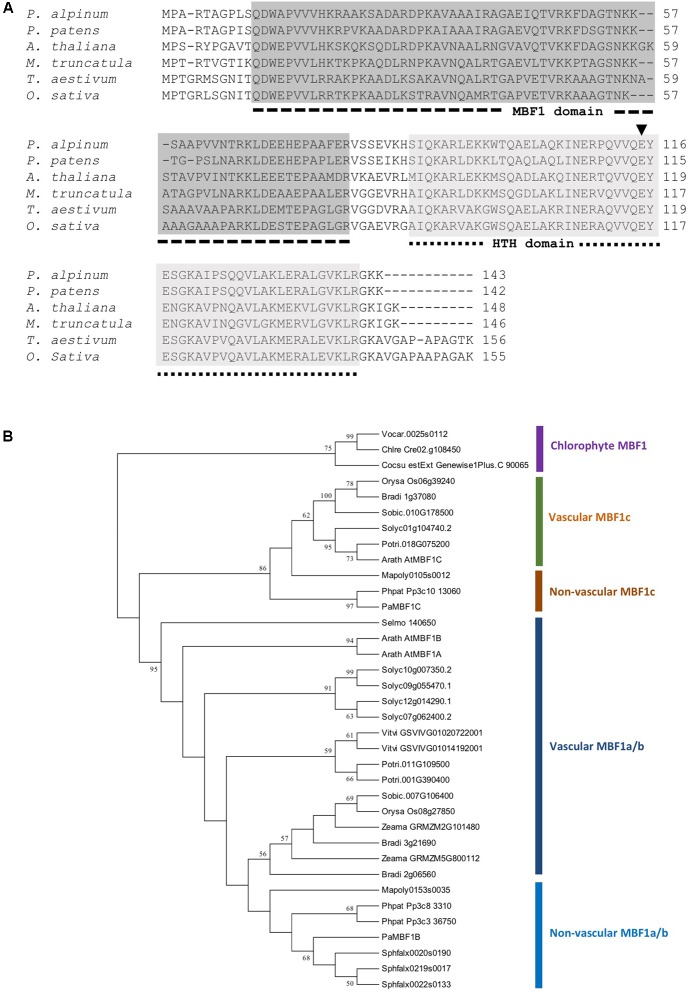
Sequence alignment and phylogenetic analysis of PaMBF1c. **(A)** Amino acid sequences of PaMBF1c protein (AJG41867) and homologs from *Physcomitrella patens* (XP_001771731), *Oryza sativa* (NP_001057974), *Medicago truncatula* (AES76734), *Triticum aestivum* (ACU43593), and *Arabidopsis thaliana* (NP_189093) were used for amino acid sequence alignment. The dotted lines below the sequence alignment indicate multiprotein bridging factor 1 (MBF1) domain and helix-turn-helix (HTH) domain. In the HTH domain, the amino acid residue glutamic acid (E) (arrow head) is conserved among all plant MBF1 proteins. **(B)** Phylogenetic tree of MBF1 proteins from diverse species. MBF1 proteins from mosses (*Polytrichastrum alpinum*; Phpat, *Physcomitrella patens*; Sphfalx, *Sphagnum fallax*; Mapoly, *Marchantia polymorpha*), Lycophyte (Selmo, *Selaginella moellendorffii*), algae (Chlre, *Chlamydomonas reinhardtii*; Cocsu, *Coccomyxa subellipsoidea*; Vocar, *Volvox carteri*), monocots (Zeama, *Zea mays*; Bradi, *Brachypodium distachyon*; Orysa, *Oryza sativa*; Sobic, *Sorghum bicolor*), and dicots (Arath, *Arabidopsis thaliana*; Vitvi, *Vitis vinifera*; Solyc, *Solanum lycopersicum*; Potri, *Populus trichocarpa*) were included. The phylogenetic tree was constructed using the Neighbor-Joining method.

The *Polytrichastrum alpinum* DNA sequence encoding multi-protein bridging factor 1c (MBF1c) protein was retrieved from our unpublished transcriptome data, based on its sequence homology with *AtMBF1c*. The gene was designated *PaMBF1c* and its sequence was submitted to GenBank (Accession number, KM978992). The total length of the 432 nucleotide coding sequence comprise of 143 deduced amino acids with an estimated molecular mass of 15.7 kDa and an isoelectric point of 10.08 predicted by ExPasy bioinformatics tools for protein structure analysis^5^. It contains two distinctive conserved domains – an MBF1 domain at the N-terminal region and a helix-turn-helix (HTH) domain at the C-terminal region (**Figure 1A**). *PaMBF1c* shares 86% identity with its homolog from *Physcomitrella patens* (XP_001771731), 64% with *Medicago truncatula* (AES76734), 66% with *Arabidopsis thaliana* (NP_189093), 59% with *Triticum aestivum* (ACU43593), and 59% identity with *Oryza sativa* (NP_001057974). Sequence alignment analysis of PaMBF1c also suggests the presence of glutamic acid (E) at the 115th amino acid position in the HTH domain (**Figure 1A**). This particular amino acid is reportedly conserved across the plant species and is essential for the binding to TATA-box binding protein (Liu et al., 2007).

The PaMBF1c protein sequence was queried against proteome datasets of various plants and chlorophytes in Phytozome^6^ to better understand the phylogenetic relations of MBF1 family proteins in plants. The phylogenetic analysis demonstrated a clear divergence of MBF1c proteins from other MBF1 family proteins (MBF1a/b) (**Figure 1B**). Interestingly, algae have only one MBF1 gene while most land plants contain at least two types MBF1 genes (**Figure 1B**), suggesting a gene duplication event early in the evolution of land plant. Within the MBF1c clade, MBF1c orthologs of all non-vascular plant species were clustered together away from those of vascular plant (**Figure 1B**) implying MBF1c differentiation among land plants.

### Expression of *PaMBF1c* Gene in *P. alpinum* under Abiotic Stress

**FIGURE 2:**
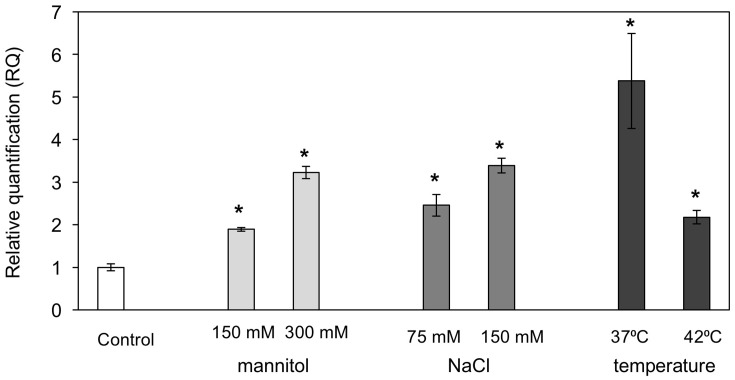
Expression of *PaMBF1c* in *P. alpinum* under various abiotic stress conditions. The *PaMBF1c* expression levels were measured by quantitative real-time PCR with total RNA from *P. alpinum* gametophores under osmotic stress (150 mM or 300 mM mannitol for 6 h), salt stress (75 or 150 mM NaCl for 6 h) or heat stress (37 or 42°C for 2 h). The *P. alpinum* tubulin gene was used as an internal control for normalization. The expression level of *PaMBF1c* grown on normal BCD was used as a control (calibrator for quantification) and was assumed as 1. Error bars represents standard deviation of means (*n* = 3). Asterisks indicate statistical significance in LSD test (*p* < 0.05).

A number of *MBF1* group genes in various species were reported to be differentially induced by various abiotic stresses (Rizhsky et al., 2002; Tsuda and Yamazaki, 2004; Kim et al., 2007). We examined *PaMBF1c* transcript abundance under salt, osmotic, and heat stress conditions in *P. alpinum*. To this end, quantitative real-time PCR (qRT-PCR) analysis was carried out using RNA isolated from *P. alpinum* gametophores treated with NaCl (75 mM or 150 mM for 6 h), mannitol (150 or 300 mM for 6 h), or high temperature (37 or 42°C for 2 h). The results revealed that *PaMBF1c* transcript levels were increased in response to various abiotic stress treatments (**Figure 2**); thus, we concluded that *PaMBF1c* is a heat, salt, and osmotic stress-responsive gene in *P. alpinum*.

### Generation and Growth Phenotype of *PaMBF1c* Overexpressing Lines

**FIGURE 3:**
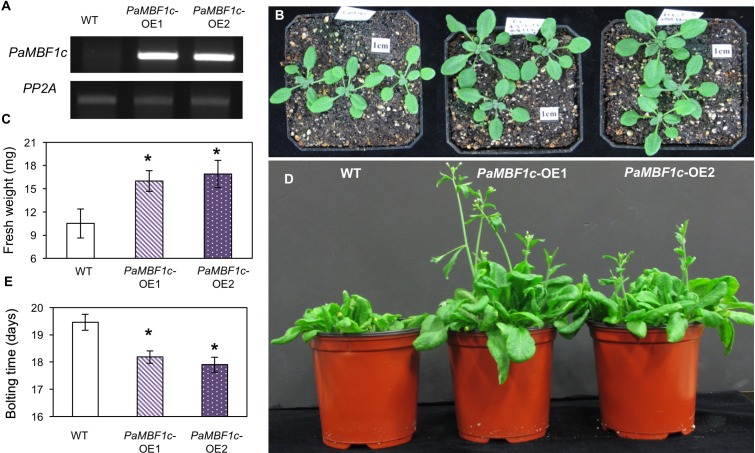
Growth and development of *PaMBF1c-*OE lines. **(A)** Higher accumulation of transgene transcripts in *PaMBF1c*-OE lines were confirmed by semi-quantitative RT-PCR. **(B)** The size of *PaMBF1c*-OE lines was bigger than WT. Pictures were taken 12 days after germination. **(C)** Measurement of fresh weights per seedling revealed a better growth of *PaMBF1c*-OE lines in comparison to WT. **(D,E)** *PaMBF1c*-OE lines bolted earlier than WT. Error bars represents standard deviation of means (*n* = 20). Asterisks indicate statistical significance in LSD test (*p* < 0.05).

To further investigate the functional roles of *PaMBF1c* in plants, we generated transgenic lines overexpressing the *PaMBF1c* gene under control of the 35S cauliflower mosaic virus promoter (35S:*PaMBF1c*). Using hygromycin resistance selection and the presence of the 35S:*PaMBF1c* transgene, we selected stable homozygous transgenic lines for 35S:*PaMBF1c* in the T4 generation (hereafter referred to as *PaMBF1c*-OE lines, where OE stands for overexpressing). Semi-quantitative RT-PCR analysis for *PaMBF1c* transcripts indeed confirmed that *PaMBF1c*-OE lines overexpressed *PaMBF1c*, as *PaMBF1c* transcripts were highly accumulated in transgenic plants (**Figure 3A**). With confirmed lines, we examined the growth and development of *PaMBF1c*-OE plants. Two-week old *PaMBF1c*-OE plants displayed better growth and development than WT plants under normal growth conditions. Fresh weight of 2-week-old transgenic plants was about 16–17 mg/seedling while that of WT was about 11 mg/seedling (**Figures 3B,C**). In addition, *PaMBF1c*-OE lines bolted 1–2 days earlier than did WT (**Figures 3D,E**).

### Evaluation of *PaMBF1c* Overexpressing Lines under Various Abiotic Stress Conditions

**FIGURE 4:**
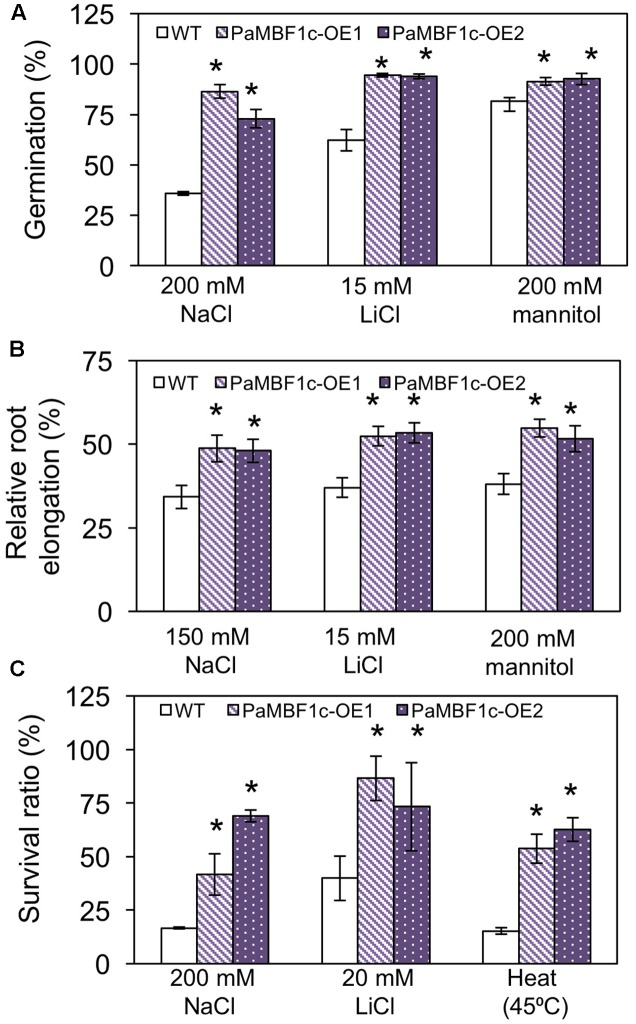
Evaluation of *PaMBF1c*-OE lines under different abiotic stress conditions. **(A)** *PaMBF1c*-OE lines germinated better than did WT under salt stress (200 mM NaCl), ionic stress (15 mM LiCl), and osmotic stress (200 mM mannitol). The number of germinated seeds was expressed as a percentage of total number of seeds planted (*n* ≥ 100). Radicle emergence was considered germination and the germination was scored after 4 days of planting. **(B)** Roots of *PaMBF1c*-OE lines elongated longer than that of WT under salt stress (150 mM NaCl), ionic stress (15 mM LiCl), and osmotic stress (200 mM mannitol). Root growth was scored after 6 days of transfer of seedlings from normal medium to stress medium. Root elongation of seedlings under stress conditions was expressed as a percentage of each stress control grown on normal MS medium after 6 days of transfer (*n* ≥ 10). **(C)** *PaMBF1c*-OE lines survived better than WT under salt stress (200 mM NaCl), ionic stress (20 mM LiCl) and heat stress (45°C for 60 min) (*n* ≥ 15). Error bars represents standard deviation of the mean values of three independent experiments. Asterisks indicate statistical significance in LSD test (*p* < 0.05).

To study the responses of *PaMBF1c*-OE plants to abiotic stress conditions, two *PaMBF1c*-OE lines from two independent transgenic events were subjected to different stress treatments. Germination of each line was tested on MS medium containing different kinds of stress agents: salt stress (200 mM NaCl), ionic stress (15 mM LiCl) or osmotic stress (200 mM mannitol). On the control medium, all lines germinated with percentages of 95–100% after 2–3 days of planting (**Supplementary Figure S1**). On mediums supplemented with 200 mM NaCl, the germination ratio of WT was decreased to 35.8%, whereas *PaMBF1c*-OE lines displayed 72.9–86.4% germination ratios (**Figure 4A**). Similarly, *PaMBF1c*-OE lines displayed 94–94.5% germination ratios on 15 mM LiCl MS medium, whereas WT germination ratio was reduced to 62.2% (**Figure 4A**). In the medium containing 200 mM mannitol, *PaMBF1c*-OE lines displayed slightly better germination than in WT (**Figure 4A**). Taken together, the results showed that *PaMBF1c*-OE lines germinate better than WT under salt, ionic, and osmotic stress.

Root growth is affected by various stress conditions and is often considered an index for stress sensitivity (Lee et al., 2006). Thus, we examined levels of stress tolerance by analyzing root growth of *PaMBF1c*-OE lines. Similarly sized seedlings (3- to 4-day-old) grown under normal condition were transferred to MS medium for salt stress (150 mM NaCl), ionic stress (15 mM LiCl), or osmotic stress (200 mM mannitol); and root elongation was measured 6 days after transfer. All lines showed similar root lengths in the control MS media (**Supplementary Figure S2**); however, relative root elongation in *PaMBF1c*-OE lines was significantly greater in the presence of 150 mM NaCl (48–48.7% vs. 34.2%), 200 mM mannitol (51.5–54.8% vs. 38.1%), or 15 mM LiCl (52.3–53.3% vs. 37%) when compared to WT (**Figure 4B**). Additionally, seedling survival was examined under stress conditions. Three- to four-day-old seedlings grown under normal conditions were transferred to MS medium supplemented with 200 mM NaCl for salt stress or 20 mM LiCl for ionic stress. For heat stress, 8-day-old seedlings grown on MS plates were heat treated at 45°C (**Figure 4C** and **Supplementary Figures S3A,B**). The *PaMBF1c*-OE lines showed significantly better survival than did WT under the presence of 200 mM NaCl (41–69% vs. 16.6%), 20 mM LiCl (73–86% vs. 40%), or heat (53–62% vs. 15%) (**Figure 4C** and **Supplementary Figures S3C–E**). We also investigated responses to cold stress in *PaMBF1c*-OE lines by measuring ion leakage after freezing stress treatment. At -6°C, both WT and *PaMBF1c*-OE showed similar ion leakage levels (**Supplementary Figure S4**); however, *PaMBF1c*-OE lines showed lower ion leakage at -9 and -12°C than WT, suggesting enhanced freezing tolerance in *PaMBF1c*-OE lines (**Supplementary Figure S4**).

### Examination of Salt Stress Tolerance in *PaMBF1c* Overexpressing Lines

**FIGURE 5:**
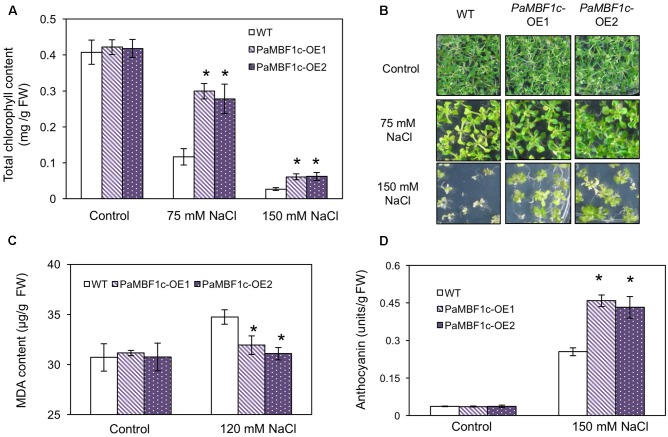
Chlorophyll, malondialdehyde (MDA), and anthocyanin levels in *PaMBF1c*-OE lines. **(A,B)** *PaMBF1c*-OE lines retained more chlorophyll content than WT at 75 and 150 mM NaCl. **(C)** *PaMBF1c*-OE lines showed lower MDA level than WT. MDA levels were examined with 21-day-old seedlings grown on MS plates with 0 or 120 mM NaCl. **(D)** *PaMBF1c*-OE lines accumulated higher amount of anthocyanin than WT. Anthocyanin content was measured with 10-day-old seedlings grown on MS plates with 0 or 150 mM NaCl. Error bars represents standard deviation of mean values of at least three independent experiments (*n* = 25 seedlings per each treatment). Asterisks indicate statistical significance in LSD test (*p* < 0.05).

We examined in detail the tolerance phenotypes of *PaMBF1c*-OE under salt stress by analyzing chlorophyll content, lipid peroxidation level, and anthocyanin accumulation. Chlorophyll (Chl) degradation is among the manifestations caused by salt and osmotic stress (Claeys et al., 2014; Alavilli et al., 2016). To further examine the effect of salt stress, we measured Chl content in WT and *PaMBF1c-*OE lines with or without salt stress. No notable differences were observed between WT and *PaMBF1c*-OE grown under control condition (**Figures 5A,B**); however, stress treatment with 75 mM or 150 mM NaCl showed that *PaMBF1c*-OE lines maintained higher Chl content than that of WT, as also evidenced by visual phenotypes (i.e., leaf bleaching) (**Figures 5A,B**). Abiotic stresses usually lead to lipid peroxidation of cell membranes which can cause irreversible damage to its functionality. MDA is considered an indicator of lipid peroxidation level (Heath and Packer, 1968). After salt stress (120 mM NaCl), the MDA content of *PaMBF1c*-OE lines was significantly lower in than that of WT, which suggests lower lipid peroxidation in *PaMBF1c*-OE (**Figure 5C**). Anthocyanin accumulation is known to be associated with improved drought and salt stress tolerance (Oh et al., 2011; Nakabayashi et al., 2014). Accordingly, 10-day-old *PaMBF1c*-OE lines grown under salt stress medium (150 mM NaCl) exhibited higher anthocyanin accumulation than WT (**Figure 5D** and **Supplementary Figure S5**). Taken all together, these results clearly demonstrated the higher salt-stress tolerance of *PaMBF1c*-OE lines than WT.

### Comparison of Stress Tolerance between *PaMBF1c* and *AtMBF1c* Overexpressing Lines

**FIGURE 6:**
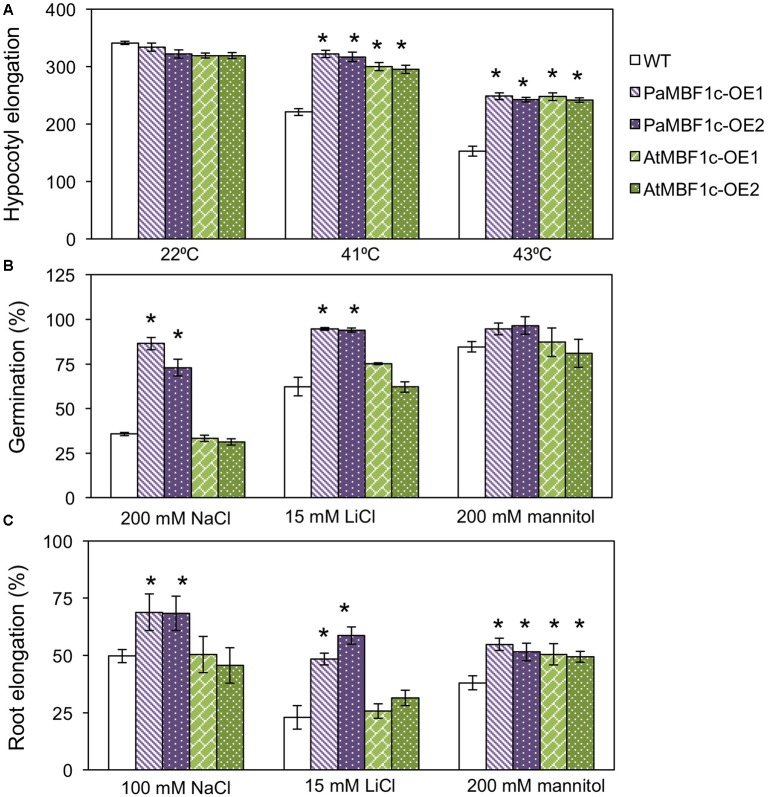
Comparison of stress tolerance between *PaMBF1c*-OE lines and *AtMBF1c*-OE lines under various abiotic stress conditions. **(A)** Comparison of hypocotyl elongation between *PaMBF1c*-OE and *AtMBF1c*-OE lines under heat stress. Both OE lines showed similar levels of heat tolerance but higher than WT (*n* ≥ 10). **(B)** Comparison of germination between *PaMBF1c*-OE and *AtMBF1c*-OE lines under salt stress (200 mM NaCl), ionic stress (15 mM LiCl), and osmotic stress (200 mM mannitol). *PaMBF1c*-OE displayed higher germination ratio than WT and *AtMBF1c*-OE lines under salt and ionic stresses (*n* ≥ 100). **(C)** Comparison of root elongation between *PaMBF1c*-OE and *AtMBF1c*-OE lines under salt stress (100 mM NaCl), ionic stress (15 mM LiCl), and osmotic stress (200 mM mannitol). *PaMBF1c*-OE showed higher root elongation than *AtMBF1c*-OE and WT under salt and ionic stresses (*n* ≥ 10). Error bars represents standard deviation of the mean values of three independent experiments. Asterisks indicate statistical significance in LSD test (*p* < 0.05).

*AtMBF1c* overexpression in Arabidopsis resulted in improved heat and osmotic stress tolerance (Suzuki et al., 2005). *AtMBF1c*-OE lines appeared to exhibit tolerance at only low levels of salt stress (50 mM NaCl) when compared to WT (Suzuki et al., 2005). Thus, we compared the stress tolerance phenotypes of our *PaMBF1c*-OE lines and the *AtMBF1c*-OE lines under our experimental conditions. We first assessed heat tolerance with two independent lines for each overexpressor. The hypocotyl elongation assay makes use of the characteristic heat inhibition of hypocotyl elongation (Hong and Vierling, 2000). *PaMBF1c*-*OE* lines showed higher hypocotyl elongation than did WT at both 41 and 43°C (**Figure 6A**), suggesting hyposensitivity to heat stress in *PaMBF1c*-OE. Similarly, *AtMBF1c*-OE lines displayed higher hypocotyl elongation when compared to WT in agreement with previous reports (Suzuki et al., 2005). Hypocotyl lengths of both OE lines were comparable after heat treatment suggesting that both *PaMBF1c* and *AtMBF1c* have similar levels of increased heat tolerance (**Figure 6A**).

We further extended the comparison to other types of stress. We treated *PaMBF1c-*OE and *AtMBF1c-*OE lines with salt (100–200 mM NaCl), ionic (15 mM LiCl) and osmotic stress (200 mM mannitol) at germination and post-germination stages. Under salt stress, *PaMBF1c*-OE lines consistently displayed higher germination ratios than WT, whereas *AtMBF1c*-OE lines germinated at almost the same ratio as WT (**Figure 6B**). In addition, the germination of *PaMBF1c*-OE lines was better than those of WT and *AtMBF1c*-OE lines under ionic stress (15 mM LiCl) (**Figure 6B**). However, germination of each line at osmotic stress (200 and 400 mM mannitol) were largely comparable, with one *PaMBF1c*-OE line demonstrating a slightly higher germination ratio than the other genotypes (**Figure 6B** and **Supplementary Figure S6**). These results suggest that *PaMBF1c*-OE performs better in terms of germination than WT or even *AtMBF1c*-OE, particularly under salt and ionic stress.

Root growth comparisons also revealed that the root of *PaMBF1c*-OE lines grew longer under salt and ionic stresses than that of both WT and *AtMBF1c*-OE (**Figure 6C**). *AtMBF1c*-OE lines exhibited root growth similar to that of WT under salt and ionic stresses (**Figure 6C**). Under osmotic stress (200 mM mannitol), root elongation was similar between *PaMBF1-*OE and *AtMBF1c*-OE lines but was significantly enhanced in comparison with WT (**Figure 6C**). Improved osmotic stress tolerance in the *AtMBF1c*-OE line was consistent with previous reports (Suzuki et al., 2005). These results revealed that except for germination under osmotic stress, *PaMBF1c*-OE lines showed enhanced tolerance at both germination and post-germination stage under all tested conditions including stress conditions in which *AtMBF1c* did not show increased tolerance. Conclusively, these results implicate *PaMBF1c* as an *MBF1c* allele which affords better coping against multiple stresses than *AtMBF1c*.

### Transcriptome Analysis of *PaMBF1c* Overexpressing Lines under Salt Stress Conditions

**FIGURE 7:**
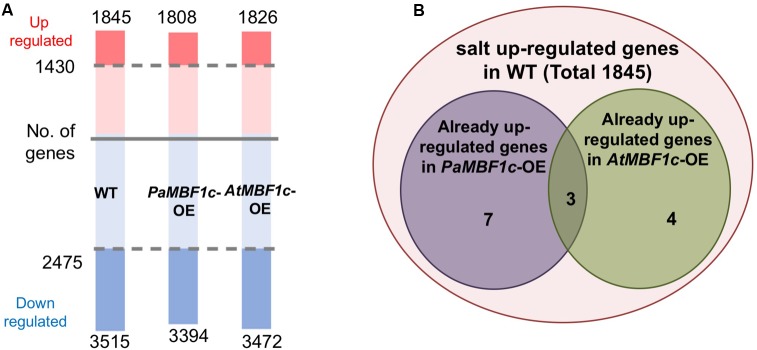
Transcriptome analysis of WT, *PaMBF1c*-OE, and *AtMBF1c*-OE lines. **(A)** Numbers of salt-regulated genes (*p*-value < 0.05, FDR corrected *p*-value < 0.05, absolute value of fold change > 1.5) in WT, *PaMBF1c*-OE, and *AtMBF1c*-OE lines. Shaded regions indicate genes commonly regulated by salt in all three lines. Numbers above the pink bars denote the total number of up-regulated genes and ones below the blue bars indicate the total number of down-regulated genes in each genotype. Numbers by the shaded regions in the middle of the bar are the total number of genes that were commonly up-/down-regulated in all variants. **(B)** Venn diagram showing 10 salt up-regulated genes that were already up-regulated in *PaMBF1c*-OE and 7 genes in *AtMBF1c*-OE plants under normal conditions.

In order to understand the gene expression patterns of *PaMBF1c*-*OE* lines in salt stress response and identify genes important for *PaMBF1c*-specific stress tolerance, we carried out RNA-Seq analysis. After sequencing with total RNA from salt-treated samples, we first defined salt-regulated genes in WT as the genes with statistically altered expression in WT under salt stress. A total of 5,360 genes were salt-regulated with 1,845 genes up-regulated and 3,515 down-regulated (**Figure 7A** and Supplementary Table S2). Among these salt-regulated genes, we then identified the up- or down-regulated genes in *PaMBF1c*-OE and *AtMBF1c*-OE lines under normal condition. We found that 10 genes in the *PaMBF1c*-OE line were already up-regulated, which included three genes that were also already up-regulated in the *AtMBF1c*-OE line (**Table 1** and **Figure 7B**). We validated the expression of several genes in **Table 1** by real-time PCR and found that gene expression of all tested genes was consistent with RNA-Seq results (**Supplementary Figure S7**).

**Table 1:** Salt up-regulated genes that were already up-regulated in the *PaMBF1*c-OE lines under normal conditions.

Gene locus	Annotation	*PaMBF1c*-OE vs. WT under normal conditions		Salt treated vs. normal in WT	
		Fold change	*p*-value	Fold change	*p*-value
At1g33480	RING/U-box superfamily protein	22.29	0	6.93	3.2.E - 05
At5g59310	Lipid transfer protein 4 (LTP4)	14.47	5.4.E - 03	542.72	0
At5g26970	Unknown protein	5.61	4.2.E - 02	7.70	8.3.E - 04
At5g51720^∗^	NEET group protein	2.29	4.9.E - 04	1.99	1.1.E - 03
At2g14610^∗^	Pathogenesis related gene1 (PR1)	2.13	1.6.E - 06	2.49	3.4.E - 12
At1g77120^∗^	Alcohol dehydrogenase 1 (ADH1)	1.98	2.3.E - 04	14.99	0
At1g29395	COR414-TM1	1.68	1.8.E - 02	4.04	0
At1g23130	Bet vl allergen family protein	1.62	0	1.53	6.2.E - 14
At4g25100	Fe-superoxide dismutase1 (FeSOD1)	1.57	0	2.27	0
At1g56580	Smaller with variable branches (SVB)	1.53	8.5.E - 07	3.52	0

*^∗^Genes already up-regulated also in *AtMBF1c*-OE under normal conditions. The list of genes shown were determined from a cut-off value (*p*-value < 0.05, corrected *p*-value of FDR < 0.05, fold change > 1.5).*

None of the salt down-regulated genes were found already down-regulated in either *PaMBF1c* or *AtMBF1c* overexpressing lines (data not shown). The already up-regulated genes, particularly *PaMBF1c*-OE specific genes, might elicit quicker response to salt stress in *PaMBF1c*-OE lines, resulting in enhanced salt tolerance in overexpressors. Among the already up-regulated genes in *PaMBF1c*-OE, the *FeSOD1* gene (At4g25100) was identified in a transcriptome comparison study by Taji et al. (2004) in which 77 genes were identified that showed higher expression under normal conditions in a halophyte *Thellungiella halophila* than in Arabidopsis. This suggests that early establishment of *FeSOD1*-mediated detoxification of reactive oxygen species (ROS) in *PaMBF1c*-OE might be an important step in the enhanced tolerance to salt stress which usually causes secondary oxidative stress.

We also compared profiles of salt-regulated genes from WT, *PaMBF1c*-OE and *AtMBF1c*-OE lines. The two overexpressors showed similar numbers of genes with altered expression under salt stress as did wild type (1,808 and 1,826up-regulated genes in *PaMBF1c*-OE and *AtMBF1c*-OE, respectively; 3,394 and 3,472 down-regulated genes in *PaMBF1c*-OE and *AtMBF1c*-OE, respectively) (**Figure 7A** and Supplementary Table S3). Expression of the majority of these genes was commonly regulated by salt stress in all genotypes; i.e., WT, *PaMBF1c*-OE, and *AtMBF1c*-OE lines each possessed the same set of 1,430 up-regulated and 2,475 down-regulated genes out of a total of 1845, 1,808 and 1,826 up-regulated and 3515, 3,394 and 3,472 down-regulated genes in WT, *PaMBF1c*-OE and *AtMBF1c*-OE, respectively (**Figure 7A** and Supplementary Table S3). At least in part, the genes with altered expression unique to *PaMBF1c*-OE might contribute to the enhanced salt tolerance of the *PaMBF1c*-OE lines.

**FIGURE 8:**
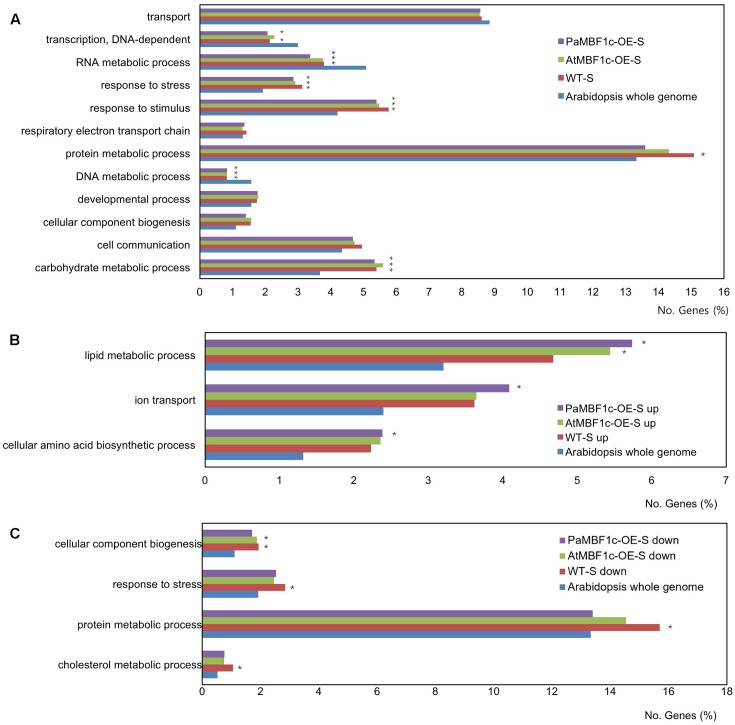
Gene ontology enrichment analysis of salt regulated genes in WT, *PaMBF1c*-OE, and *AtMBF1c*-OE lines. **(A)** Gene Ontology (GO) terms with significance (corrected *p*-value of Bonferroni correction < 0.05) from the GO enrichment analysis with salt-regulated genes using Arabidopsis whole genome as a comparison reference. **(B)** Functional GO classification of genes salt up-regulated genes based on plant GO slim terms. **(C)** Functional GO classification of genes salt down-regulated genes based on plant GO slim terms. Only GO terms with significance (corrected *p*-value of Bonferroni correction < 0.05) from the GO enrichment analysis were presented. For suffix of each line in the graph legends, “-S” indicates “salt-treated,” “up” and “down” mean “up-regulated” and “down-regulated,” respectively. Asterisks indicate statistical significance.

Gene ontology enrichment analysis within the category ‘biological processes’ was conducted with salt-regulated genes using the whole Arabidopsis genome set as reference. The overall distribution pattern of salt-regulated genes in GO terms was well conserved among WT, *PaMBF1c*-OE, and *AtMBF1c*-OE lines under salt stress conditions (**Figure 8A**). The terms RNA metabolic process, response to stress, response to stimulus, DNA metabolic process, and carbohydrate metabolic process were significantly over- or under-represented in all three plants compared to the Arabidopsis genome (*p*-value of the Bonferroni correction for multiple testing < 0.05) (**Figure 8A**). The term protein metabolic process was significantly over-represented only in WT plants but not in *PaMBF1c*-OE or *AtMBF1c*-OE, suggesting that protein metabolism is a major biological process affected in WT plants under salt stress.

To increase the resolution of GO enrichment analysis, we divided salt-regulated genes into salt up-regulated and salt down-regulated groups and conducted separate GO enrichment analyses for each with the whole Arabidopsis genome as reference. For salt up-regulated genes, GO terms in biological processes that were significantly over-represented in *PaMBF1c*-OE plants were ‘lipid metabolic process,’ ‘ion transport,’ and ‘cellular amino acid biosynthetic process’ (**Figure 8B**). The corresponding salt up-regulated genes included in these terms were listed in Supplementary Table S4. Given the possibility that improved salt tolerance in *PaMBF1c*-OE might be mainly due to enhanced ionic stress tolerance, we manually surveyed genes for the term ‘ion transport’ that were significantly up-regulated in *PaMBF1c*-OE only. Notably, genes involved in ATP production and transport (*Mitochondrial phosphate transporter* [At3g48850] and *ADP/ATP carrier 3* [At4g28390]) as well as ATP-dependent Ca^2+^ pumping (*ECA1* [At1g07810]) were among the *PaMBF1c*-OE specific salt up-regulated genes (Supplementary Table S4) (Liang et al., 1997; Wu et al., 2002). Thus, enhanced salt tolerance in *PaMBF1c*-OE might be caused in part by promotions of ATP synthesis and Ca^2+^ signaling.

For salt down-regulated genes, the GO terms ‘cellular component biogenesis,’ ‘response to stress,’ ‘protein metabolic process,’ and ‘cholesterol metabolic process’ were significantly over-represented in WT plants under salt-stress condition but not so in *PaMBF1c*-OE or *AtMBF1c*-OE plants with the exception of ‘cellular component biogenesis’ which was over-represented in both WT and *AtMBF1c*-OE plants (**Figure 8C**). Salt down-regulated genes in these GO terms were listed in Supplementary Table S5. We reasoned that among the genes of these GO terms, those that are either salt up-regulated or not down-regulated only in *PaMBF1c*-OE lines would be more highly associated with enhanced salt stress tolerance in *PaMBF1c*-OE. And thus, we manually surveyed those groups of genes. Among the interesting findings that we noticed was that many ribosome and translation-related genes were not salt down-regulated only in *PaMBF1c*-OE (Supplementary Table S5), which suggests that *PaMBF1c* might function in promoting protein synthesis processes particularly to acquire enhanced salt stress tolerance.

## Discussion

Developing crop plants with inbuilt tolerance for multiple stresses is a requisite for mitigating damage to global agriculture productivity. So far, numerous efforts have been made to clone novel genes from a diverse array of species acclimated to various adverse environments. Our study demonstrated that ectopic expression of *PaMBF1c* enhanced the adaption of transgenic Arabidopsis to various abiotic stresses and in particular, to salt stress.

Phylogenetic analysis of PaMBF1c and MBF1s from other plant species ranging from algae to dicot plants suggested a clear divergence between MBF1c and MBF1a/b (MBF1a and MBF1b) proteins among both vascular and non-vascular plants (**Figure 1B**). Other studies have also concluded the same (Tsuda and Yamazaki, 2004; Liu et al., 2007). This denotes that MBF1c proteins of plants might possess non-redundant functions which differ from those of MBF1a/b proteins despite sharing similar conservative domains among the MBF1 family proteins.

Another interesting finding was the general maintenance of *MBF1c* as a single gene versus the variability of *MBF1a* or *MBF1b* group genes depending on species (**Figure 1B**). Phylogenetic analysis revealed that this divergence between *MBF1a/b* and *MBF1c* is apparent in both vascular and non-vascular plants. Algae species (i.e., *Chlamydomonas reinhardtii, Coccomyxa subellipsoidea*, and *Volvox carteri*) contain only one *MBF1* gene while most land plants possess two (*MBF1a/b* and *MBF1c*), suggesting that gene duplication occurred early in land plant emergence. In some species such as *Marchantia polymorpha, Oryza sativa*, and *Sorghum bicolor*, only two copies of *MBF1* genes (one *MBF1a* or *MBF1b*, and one *MBF1c*) exist. Species such as *A. thaliana*, *Populus trichocarpa*, *Physcomitrella patens*, and *Brachypodium distachyon* appeared to have experienced a single round of recent gene duplication in the *MBF1a*/*b* gene which resulted in three *MBF1* genes including the *MBF1c* gene.

Thus, it is likely that *MBF1c* is functionally distinct from *MBF1a/b* since its evolution and mainly function in stress tolerance regulation. This notion has been supported by many studies including our own; *PaMBF1c*-OE demonstrated similar enhanced stress tolerance to heat and osmotic stress as *AtMBF1c*-OE (Suzuki et al., 2005). In addition, *PaMBF1c* overexpression brought about enhanced salt stress tolerance which was not observed in the case of *AtMBF1c* overexpression. Thus, *PaMBF1c* may have obtained additional function during evolution to cope with the high salt conditions of Antarctic soil (see below).

Consistent with its function, expression of *MBF1c* was highly induced by different abiotic stresses in plants. For example, transcript levels of *TaMBF1c* was increased under drought, H_2_O_2_, and heat stress conditions in *Triticum aestivum* (Qin et al., 2015). Similarly, expression of *PaMBF1c* was induced by mannitol, NaCl, and heat treatment (**Figure 2**). In Arabidopsis, expression of *AtMBF1c* was also induced in response to heat and drought or a combination of both heat and osmotic stress (Suzuki et al., 2005). In contrast, the expression of *AtMBF1a* and *AtMBF1b* was not altered by abiotic stress, but rather developmentally regulated (Tsuda and Yamazaki, 2004). These observations suggest that *MBF1* genes have evolved not only at the coding sequence level, but also at the level of regulatory sequence for the diversification of function.

In addition to their functions in abiotic stress responses, *PaMBF1c* and *AtMBF1c* appear to have some role in development. In comparison to WT, both *PaMBF1c*-OE and *AtMBF1c*-OE displayed higher fresh weight and flowered earlier. The enhanced growth by *MBF1* overexpression was also reported in a *CaMBF1* overexpression study even in cases showing stress sensitive phenotypes (Guo et al., 2014). This robust growth of *MBF1* overexpression plants might be due to elevated endoreduplication and promotion of cell expansion throughout the leaves (Tojo et al., 2009). These observations indicate the functional similarity of *PaMBF1c* and *AtMBF1c* during development. Thus, the fact that enhanced salt stress tolerance was achieved only by *PaMBF1c* overexpression, despite these similarities between *PaMBF1c* and *AtMBF1c*, underscores a broader functional spectrum of *PaMBF1c* from the polar moss.

Adverse effects of salt stress on plants are the combined result of osmotic stress and ionic stress. Ionic stress tolerance was enhanced only in *PaMBF1c*-OE at 15–20 mM LiCl, while similar levels of osmotic stress tolerance was measured in *PaMBF1c*-OE and *AtMBF1c*-OE at 200 mM mannitol (**Figure 6**). This indicates that the *PaMBF1c* gene might cope better against ionic stress imposed by salt treatment in comparison with the *AtMBF1c* gene. The moss *P. alpinum* was collected from Antarctic soils which contain characteristically high contents of soluble salts (i.e., sulfates, chlorides, nitrates, potassium, calcium, and magnesium) resulting from chemical weathering of rocks, marine salt deposits and sedimentary rock leaching (Claridge and Campbell, 1977). Thus, *PaMBF1c* might have evolved to function better in the high salt conditions of Antarctic, probably through optimization of amino acid residues. We found that there are 14 amino acid residues in PaMBF1c different from in *Triticum aestivum MBF1c* (ACU43593) and *AtMBF1c* (NP_189093) whose functions were shown to be involved in abiotic stress tolerance (Suzuki et al., 2005; Qin et al., 2015). However, we cannot rule out the possibility that foreign gene (i.e., *PaMBF1c*) overexpression could have escaped from the endogenous gene regulation system of Arabidopsis to outperform endogenous gene (i.e., *AtMBF1c*) overexpression in stress response. Still, it cannot be denied that the case of *PaMBF1c* shows a good example of the advantage of using foreign genes to improve stress tolerance in plants. A question may arise as to whether the stress tolerance phenotypes in *PaMBF1c*-OE might be due to its enhanced growth; however, bigger sized plants do not always result in improved stress tolerance. For example, *CaMBF1* overexpression resulted in Arabidopsis with large leaves but with reduced stress tolerance to cold and salt stress (Guo et al., 2014). In our *PaMBF1c*-OE lines, we did not observe enhanced root growth under normal conditions (**Supplementary Figure S2**) despite the fact that *PaMBF1c*-OE lines showed longer root length under stress conditions than WT and, in some cases, both WT and *AtMBF1c*-OE (**Figures 4**, **6**). In addition, analysis of germination, MDA content, and anthocyanin content, which normally are not directly related to enhanced growth, suggested improved salt tolerance in *PaMBF1c*-OE. It should be noted that germination, MDA content and anthocyanin content were very similar among the tested lines under control conditions. Therefore, we believe that improved stress tolerance by *PaMBF1c* overexpression is not likely due to enhanced growth.

Thus, how does *PaMBF1c*, but not *AtMBF1c*, mechanistically bring about enhanced salt stress tolerance? A simple answer to this question would be that the genes with salt-altered expression only in *PaMBF1c*-OE account for the beneficial *PaMBF1c* function. According to this view, attention needs to be given to the genes involved in the over-represented GO terms of lipid metabolic process, ion transport, and cellular amino acid biosynthetic process among salt up-regulated genes in *PaMBF1c*-OE (**Figure 8B**). Another candidate gene group for better salt tolerance in *PaMBF1c*-OE could be the salt-regulated genes that are already up-regulated under normal conditions (**Table 1**). Ten and seven salt-induced genes were already highly expressed in *PaMBF1c*-OE and *AtMBF1c*-OE under normal conditions, respectively (**Table 1** and **Figure 7B**). The 10 up-regulated genes in *PaMBF1c*-OE included three genes that were also up-regulated in *AtMBF1c*-OE (**Table 1** and **Figure 7B**).

Thus, these already up-regulated genes in *PaMBF1c*-OE might prime the OE lines for quicker responses to salt stress resulting in enhanced salt tolerance in the *PaMBF1c*-OE lines. One such rapid response could be the salt-induced removal of ROS by *FeSOD1* (At4g25100) whose expression was already high under normal conditions also in the salt cress *T. halophila* (Taji et al., 2004). It is known that salt stress induces oxidative stress (Hernández et al., 1993). Also, oxidative stress is shown to cause ATP depletion (Tiwari et al., 2002). Thus, given the fact that ATP production and transport genes (At3g48850, At4g28390) and ATP-dependent Ca^2+^ pump, *ECA1* (At1g07810) were uniquely salt up-regulated only in *PaMBF1c*-OE (Supplementary Table S4), the enhancement of salt tolerance in *PaMBF1c*-OE could involve ROS detoxification, maintenance of ATP homeostasis, and facilitation of Ca^2+^ signaling. Additionally, *PaMBF1c*-OE function in salt stress might be associated with protein synthesis, given that many ribosome and translation-related genes, which were otherwise down-regulated by salt, were shown not down-regulated solely in *PaMBF1c*-OE lines. Indeed, several studies have reported that some ribosome- and translation-related genes were shown to be involved in abiotic stress responses; for example, knock-down of eukaryotic translation initiation factor 5A (eIF5A) in Arabidopsis brought about hypersensitivity to heat, oxidative and osmotic stresses, while overexpression of eIF5A resulted in osmotic stress tolerance (Ma et al., 2010; Xu et al., 2011). In addition, Arabidopsis mutants defective in cytosolic 60S ribosomal maturation factor *REIL* displayed cold-sensitive phenotypes (Schmidt et al., 2013). Further molecular and physiological studies will be needed to uncover if and how these genes function in *PaMBF1c*-mediated salt tolerance.

## Author Contributions

HL and B-hL conceived and designed the research; HA and MP performed the experiments; HA, HL, and B-hL discussed the results and wrote the paper.

## Conflict of Interest Statement

The authors declare that the research was conducted in the absence of any commercial or financial relationships that could be construed as a potential conflict of interest.

## Footnotes

1 https://phytozome.jgi.doe.gov/pz/portal.html

2 http://www.ncbi.nlm.nih.gov/Structure/cdd/wrpsb.cgi

3 www.ncbi.nlm.nih.gov/sra/

4 http://pantherdb.org/

5 http://web.expasy.org/compute_pi

6 http://www.phytozome.net

## Supplementary Material

FIGURE S1 Germination of WT, *PaMBF1c*-OE, and *AtMBF1c*-OE lines on the control media for each stress treatment. All lines showed near 100% germination levels on the control media. The control MS/agar plates were prepared separately for each stress treatment.

FIGURE S2 Root elongation of WT, *PaMBF1c*-OE, and *AtMBF1c*-OE lines on the control media for each stress treatment. Root growth on control media (MS only) for **(A)** salt stress (NaCl), **(B)** ionic stress (LiCl) and **(C)** osmotic stress (mannitol). All lines showed similar root elongation on the control media (average of 2.56 ± 0.22 cm on 6 days after transfer). The control MS/agar plates were prepared separately for each stress treatment.

FIGURE S3 Survival test of WT and *PaMBF1c*-OE lines under different abiotic stress conditions. **(A)** Seedlings on MS/agar plates before heat stress treatment. **(B)** Seedlings on MS/agar plates after heat stress treatment (45°C for 60 min). **(C)** Seedlings on control MS/agar plates for salt and ionic stress treatments. **(D)** Seedlings on 200 mM NaCl plates (salt stress). **(E)** Seedlings on 20 mM LiCl plates (ionic stress).

FIGURE S4 *PaMBF1c*-OE lines showed lower ion leakage than WT at low temperatures. Fully expanded fourth or fifth rosette leaves with petiole from 3-week-old seedlings were used for the experiment. Ion leakage was expressed as a percentage of total electrolytes at a given temperature. Error bars represents standard deviation of the mean values of five independent experiments.

FIGURE S5 Anthocyanin accumulation under salt stress. **(A)** Ten-day-old seedlings grown on control and 150 mM NaCl plates (salt stress) **(B)** Quantification of anthocyanin accumulation in each line. Error bars represents standard deviation (*n* = 25 seedlings per each treatment). Asterisks indicate statistical significance in LSD test (*p* < 0.05).

FIGURE S6 Comparison of germination between *PaMBF1c*-OE and *AtMBF1c*-OE lines under different concentrations of mannitol. Error bars represents standard deviation of the mean values of three independent experiments.

FIGURE S7 Validation of the gene expression profiles obtained in RNA-sequencing by qRT-PCR. Relative expression levels of selected genes were determined by qRT-PCR using the cDNA synthesized from RNA isolated from the 14-day-old seedlings treated to high salt stress (300 mM NaCl for 6 h). **(A)** RING/U-box super family protein (At1g33480); **(B)** lipid transfer protein 4 (At5g59310); **(C)** Fe-superoxide dismutase (At4g25100); **(D)** hypothetical protein (At5g26970). Arabidopsis Clathrin (At4g24550) gene was used as an internal control for normalization of different cDNA samples. Three biological replicates were averaged and error bars represent standard deviation.
