# Supplementary figures and images for: Antarctic Moss Multiprotein Bridging Factor 1c Overexpression in Arabidopsis Resulted in Enhanced Tolerance to Salt Stress
Source: Front Plant Sci. 2017 Jul 11;8:1206. doi: 10.3389/fpls.2017.01206 (PMC5504242; doi:10.3389/fpls.2017.01206)

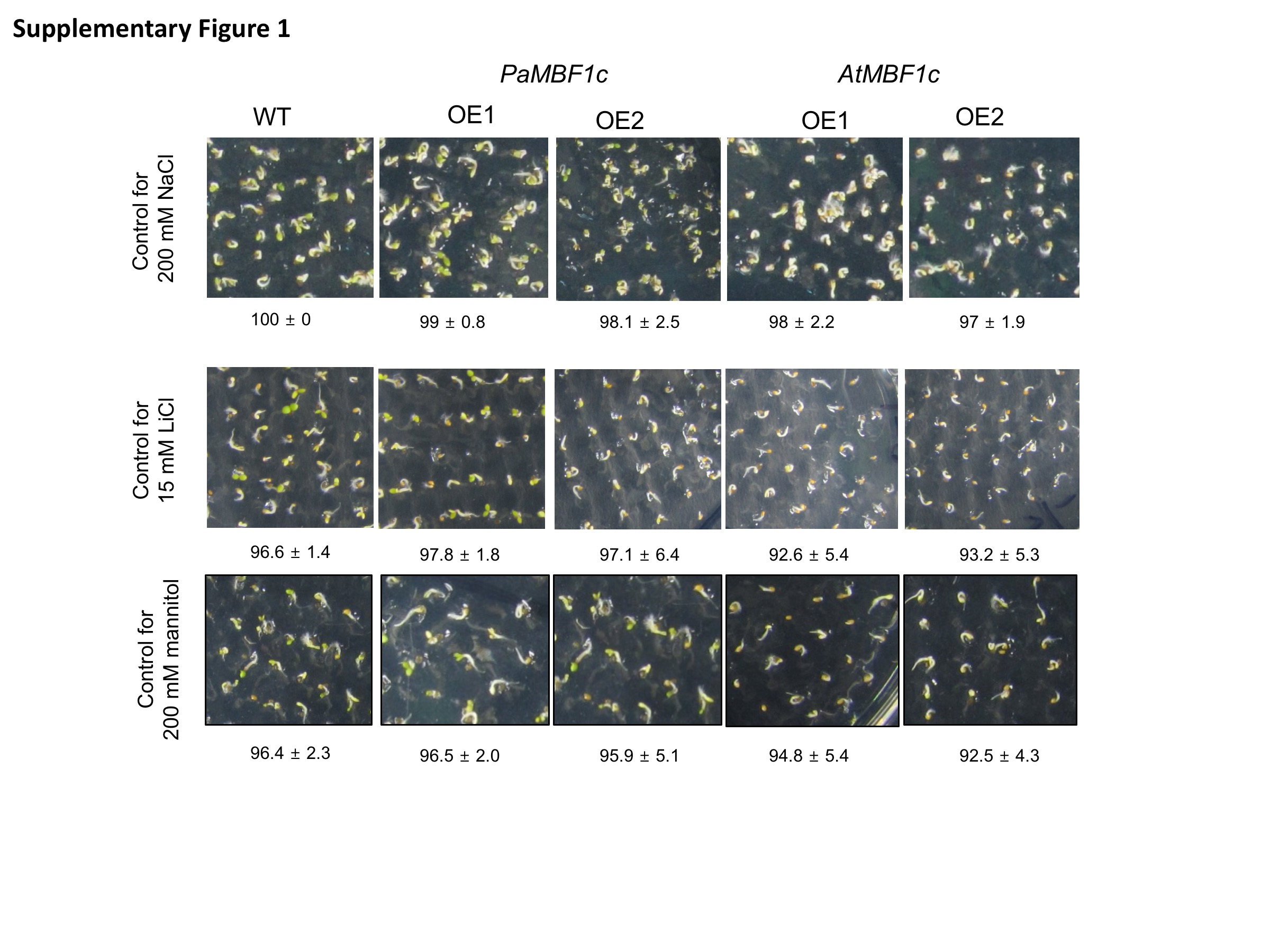

Supplement: FIGURE S1 — Germination of WT, PaMBF1c-OE, and AtMBF1c-OE lines on the control media for each stress treatment. All lines showed near 100% germination levels on the control media. The control MS/agar plates were prepared separately for each stress treatment. [file Image_1.JPEG]

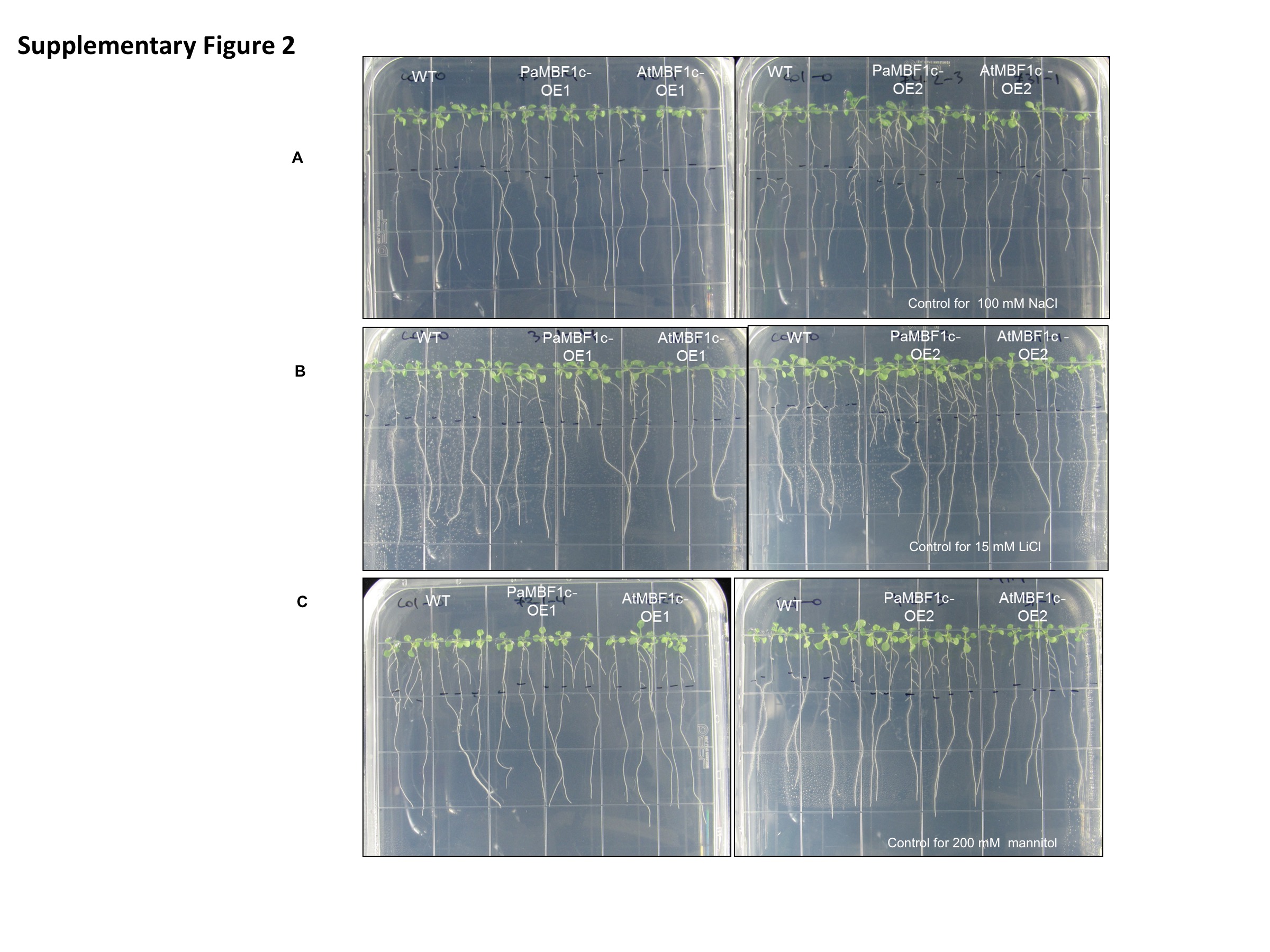

Supplement: FIGURE S2 — Root elongation of WT, PaMBF1c-OE, and AtMBF1c-OE lines on the control media for each stress treatment. Root growth on control media (MS only) for (A) salt stress (NaCl), (B) ionic stress (LiCl) and (C) osmotic stress (mannitol). All lines showed similar root elongation on the control media (average of 2.56 ± 0.22 cm on 6 days after transfer). The control MS/agar plates were prepared separately for each stress treatment. [file Image_2.JPEG]

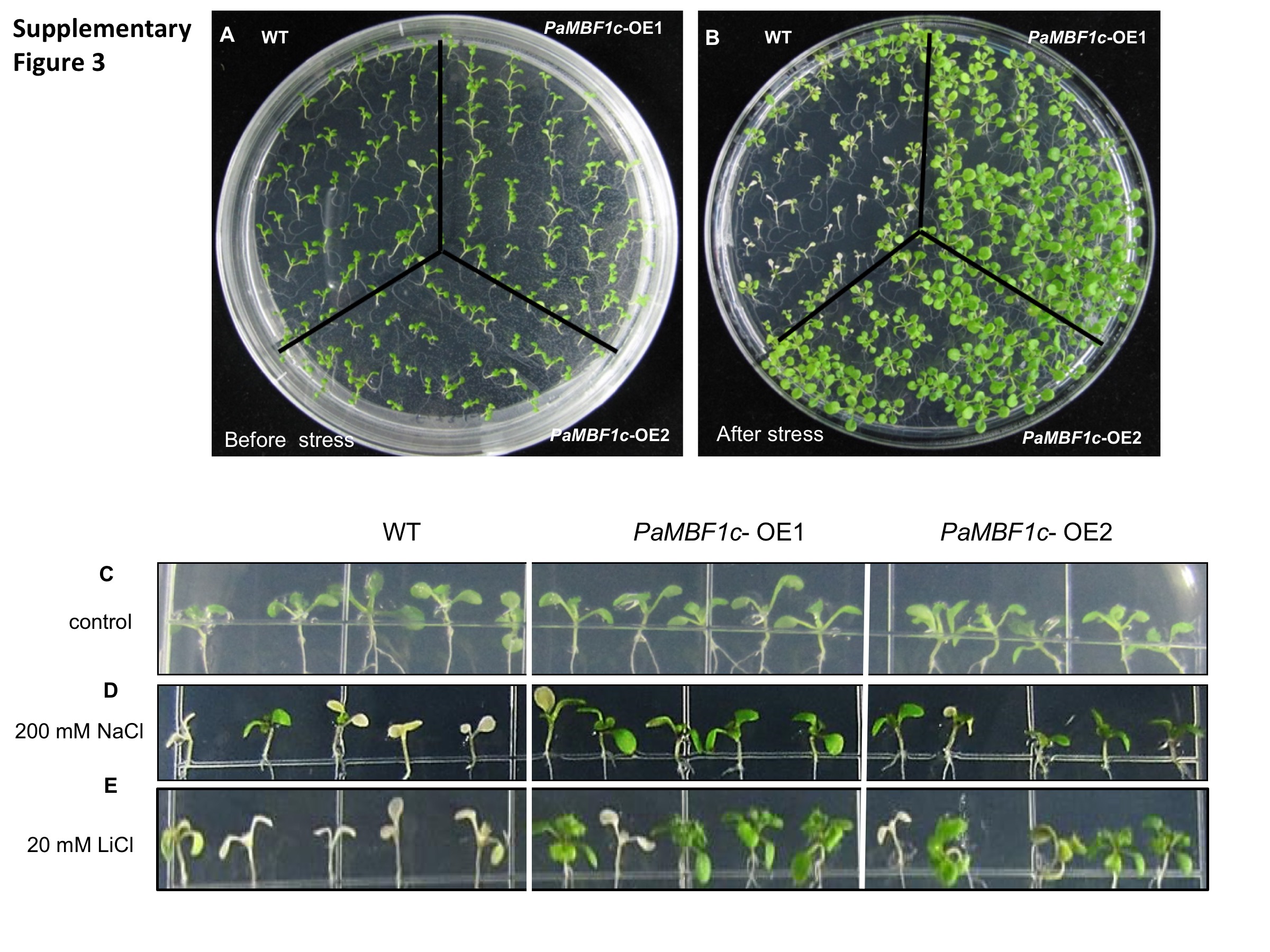

Supplement: FIGURE S3 — Survival test of WT and PaMBF1c-OE lines under different abiotic stress conditions. (A) Seedlings on MS/agar plates before heat stress treatment. (B) Seedlings on MS/agar plates after heat stress treatment (45°C for 60 min). (C) Seedlings on control MS/agar plates for salt and ionic stress treatments. (D) Seedlings on 200 mM NaCl plates (salt stress). (E) Seedlings on 20 mM LiCl plates (ionic stress). [file Image_3.JPEG]

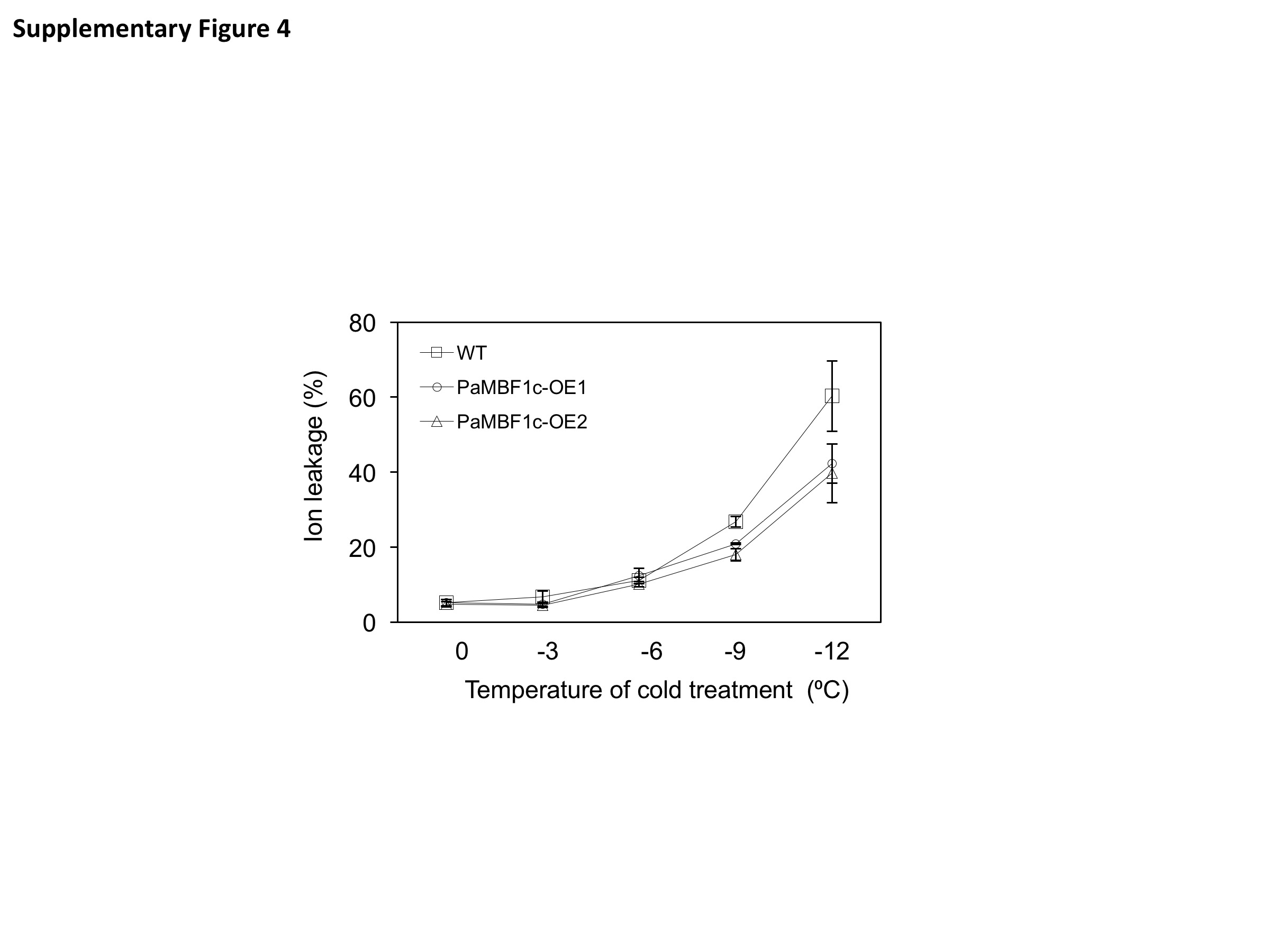

Supplement: FIGURE S4 — PaMBF1c-OE lines showed lower ion leakage than WT at low temperatures. Fully expanded fourth or fifth rosette leaves with petiole from 3-week-old seedlings were used for the experiment. Ion leakage was expressed as a percentage of total electrolytes at a given temperature. Error bars represents standard deviation of the mean values of five independent experiments. [file Image_4.JPEG]

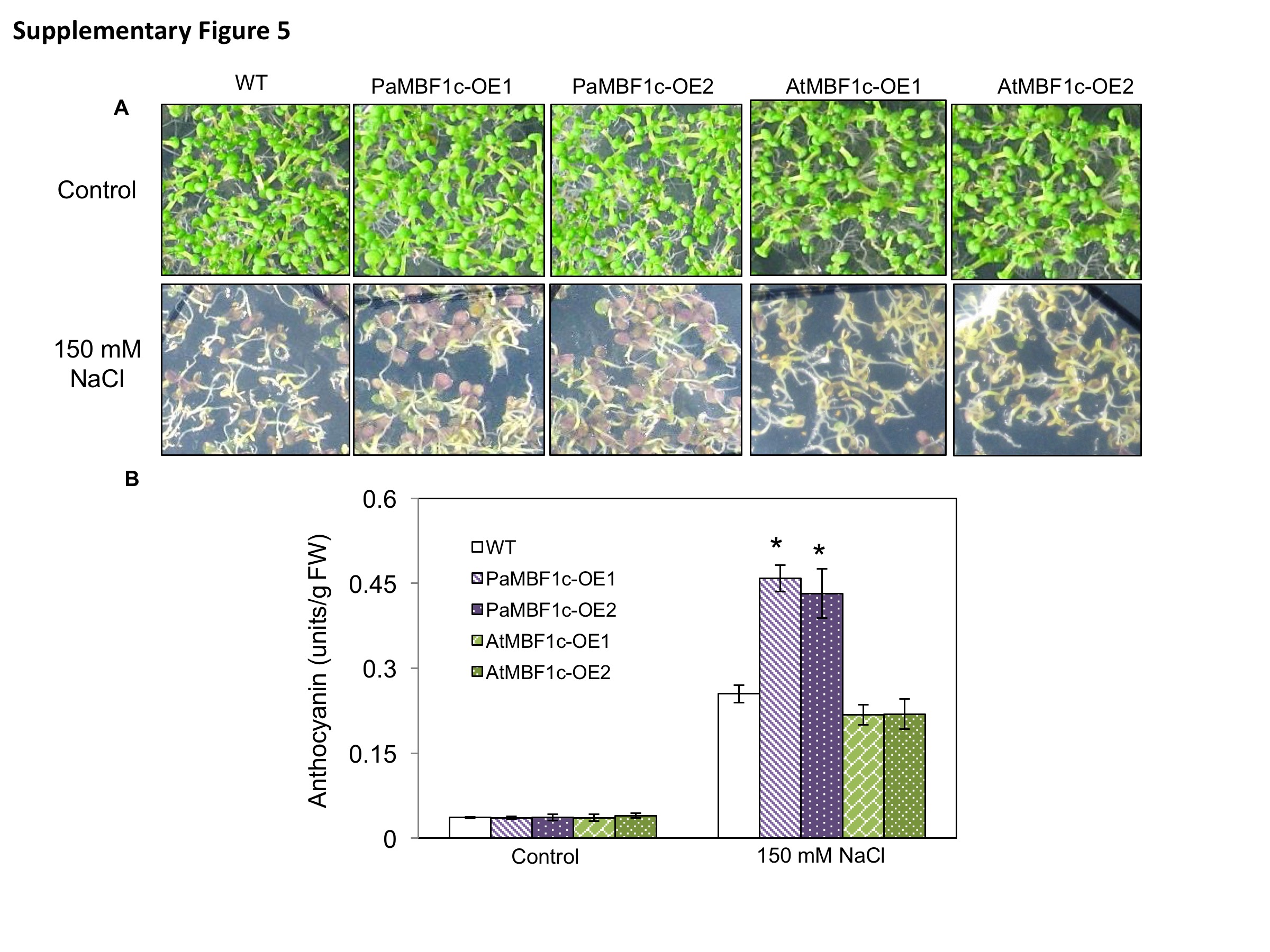

Supplement: FIGURE S5 — Anthocyanin accumulation under salt stress. (A) Ten-day-old seedlings grown on control and 150 mM NaCl plates (salt stress) (B) Quantification of anthocyanin accumulation in each line. Error bars represents standard deviation (n = 25 seedlings per each treatment). Asterisks indicate statistical significance in LSD test (p < 0.05). [file Image_5.JPEG]

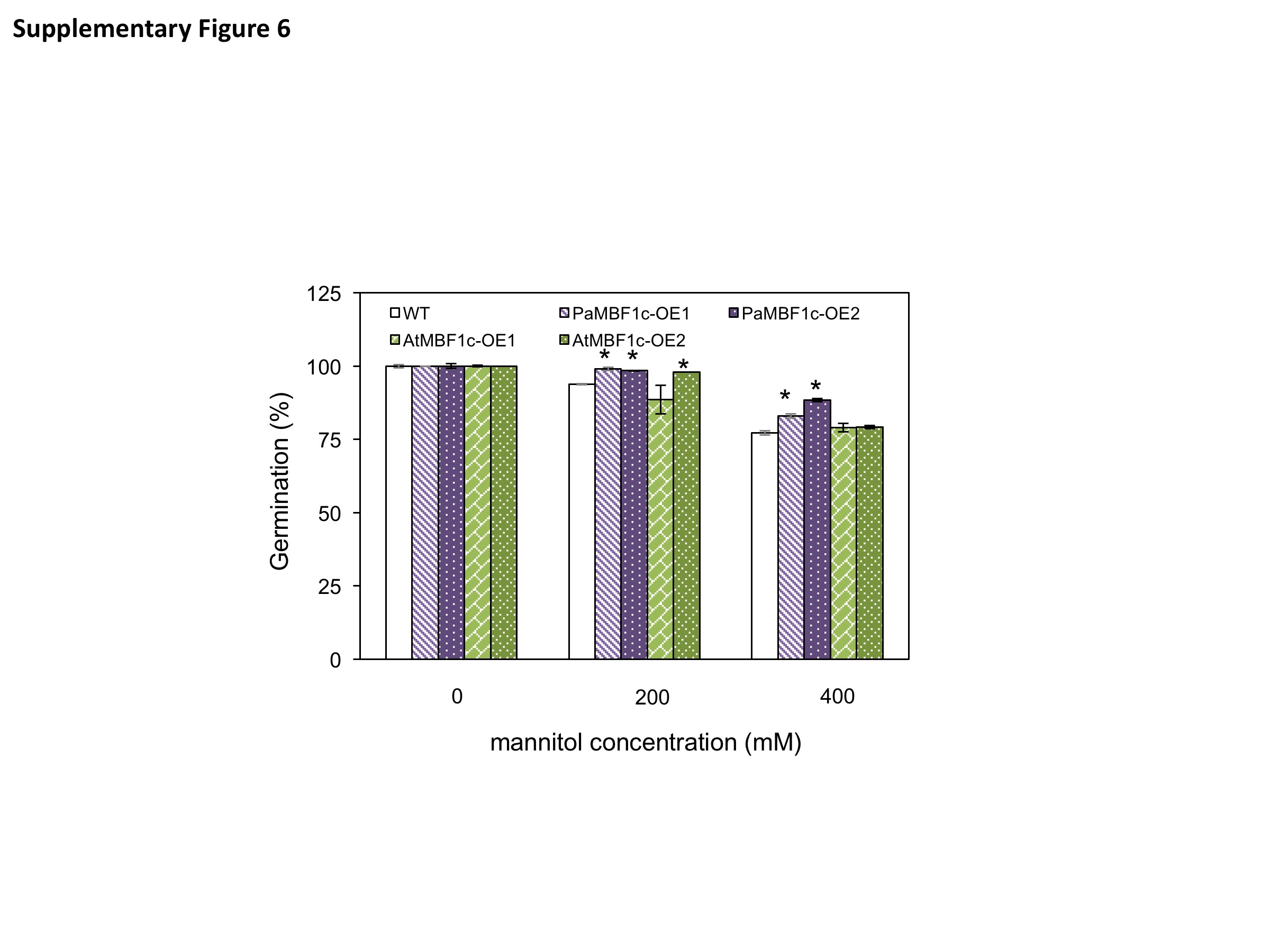

Supplement: FIGURE S6 — Comparison of germination between PaMBF1c-OE and AtMBF1c-OE lines under different concentrations of mannitol. Error bars represents standard deviation of the mean values of three independent experiments. [file Image_6.JPEG]

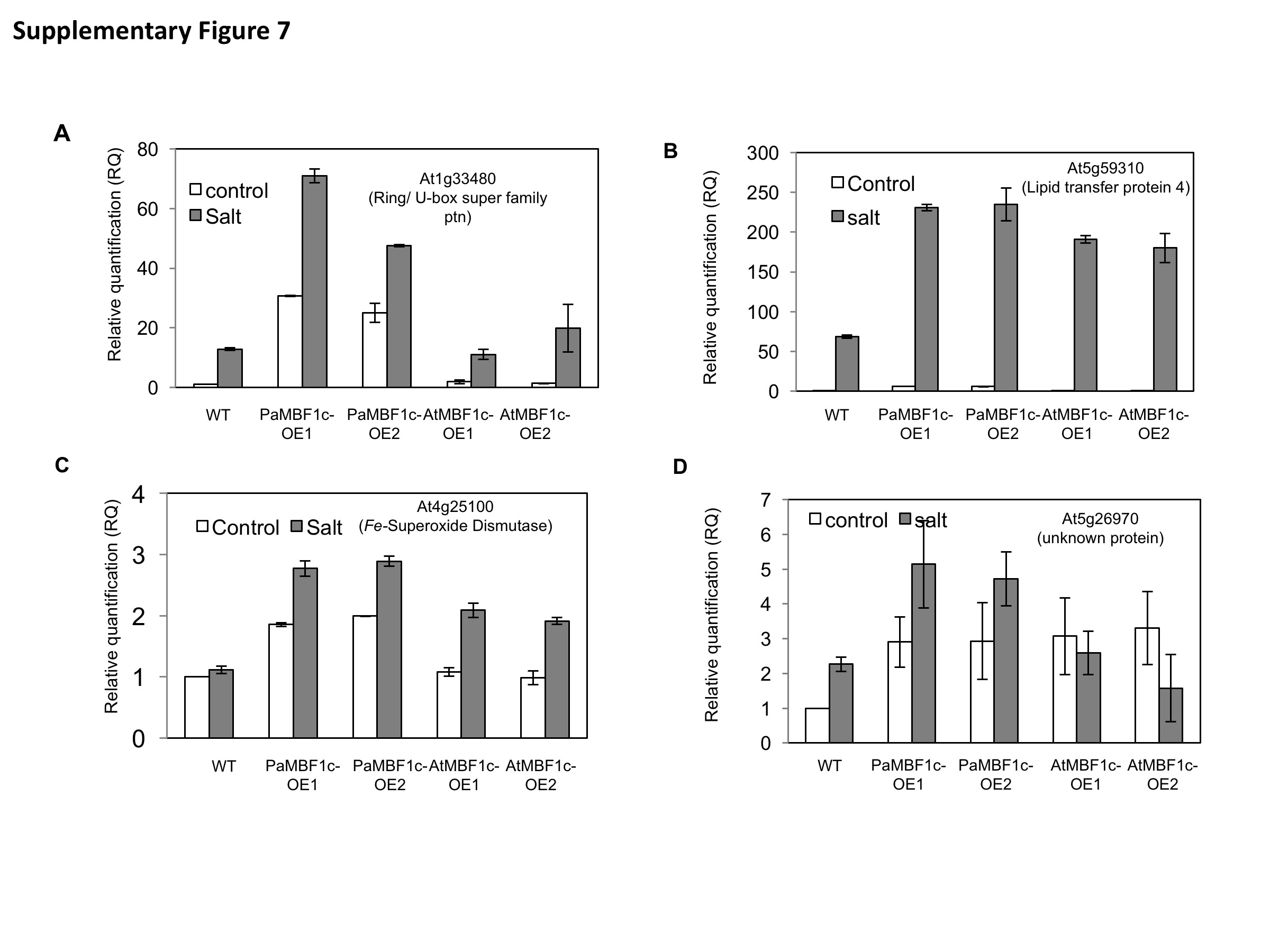

Supplement: FIGURE S7 — Validation of the gene expression profiles obtained in RNA-sequencing by qRT-PCR. Relative expression levels of selected genes were determined by qRT-PCR using the cDNA synthesized from RNA isolated from the 14-day-old seedlings treated to high salt stress (300 mM NaCl for 6 h). (A) RING/U-box super family protein (At1g33480); (B) lipid transfer protein 4 (At5g59310); (C) Fe-superoxide dismutase (At4g25100); (D) hypothetical protein (At5g26970). Arabidopsis Clathrin (At4g24550) gene was used as an internal control for normalization of different cDNA samples. Three biological replicates were averaged and error bars represent standard deviation. [file Image_7.JPEG]
